# Targeting bromodomain and extra-terminal proteins to inhibit neuroblastoma tumorigenesis through regulating MYCN

**DOI:** 10.3389/fcell.2022.1021820

**Published:** 2022-09-16

**Authors:** Xiyao Shi, Ying Wang, Longhui Zhang, Wenjie Zhao, Xiangpeng Dai, Yong-Guang Yang, Xiaoling Zhang

**Affiliations:** ^1^ Key Laboratory of Organ Regeneration and Transplantation of Ministry of Education, First Hospital, Jilin University, Changchun, China; ^2^ National-Local Joint Engineering Laboratory of Animal Models for Human Disease, First Hospital, Jilin University, Changchun, China; ^3^ Department of Laboratory Medicine, First Hospital, Jilin University, Changchun, China; ^4^ International Center of Future Science, Jilin University, Changchun, China

**Keywords:** BET family proteins, BRD4 inhibitors, neuroblastoma, epigenetic regulation, MYCN

## Abstract

Bromodomain and extra-terminal domain (BET) family proteins play important roles in regulating the expression of multiple proto-oncogenes by recognizing acetylation of histones and non-histone proteins including transcription factors, which subsequently promote tumor cell proliferation, survival, metastasis and immune escape. Therefore, BET family proteins are considered attractive therapeutic targets in various cancers. Currently, blocking of the BET proteins is a widely used therapeutic strategy for *MYCN* amplified high-risk neuroblastoma. Here, we summarized and reviewed the recent research progresses for the critical function of BET proteins, as an epigenetic reader, on tumorigenesis and the therapeutic potential of the BET/BRD4 inhibitors on *MYCN* amplified neuroblastoma. We also discussed the combined therapeutic strategies for BET inhibitor-resistant neuroblastoma.

## Introduction

Epigenetics are the hereditary changes in gene function without alterations in the DNA sequence, which eventually leads to phenotypic changes (Jeong et al., 2014; Mondal et al., 2022). Studies have shown that epigenetic regulation of oncogenes plays an important role in the expression of these genes and subsequent occurrence and development of human cancer (Hillyar et al., 2020). Furthermore, epigenetic modifications also regulate the tumor immune monitoring such as activation of T cells, generation and recognition of tumor antigens (Sun et al., 2022). Histone acetylation is one of the major epigenetic modifications. Histone acetyltransferases (HATs) is the main “writer” of histone acetylation, and histone deacetylases (HDAC) is the main “erasers” to remove acetyl groups from histone and non-histone proteins. The bromodomain and extra-terminal domain (BET) family proteins act as the “readers” of histone acetylation. Dysregulation of BET family proteins which caused a high acetylation levels promotes transcription of multiple oncogenes and participates in the occurrence and development of inflammation and cancers (Anand et al., 2013; Huang et al., 2017; Otto et al., 2019). Given the critical function of BET proteins in regulating oncogenes expression and cell proliferation, they are now considered attractive therapeutic targets in many cancer types (Chung et al., 2011). Currently, studies have shown that BET protein inhibitors exhibit significant anti-tumor effects *in vitro* and *in vivo*. A plenty of BET family protein inhibitors, such as RVX-208, I-BET 762, OTX 015, CPI0610, and TEN-010, are developed and now in the clinical trials (Herait et al., 2014; Fu et al., 2015). However, the emerged drug resistance limited the wide application of BET inhibitors and it was reported that the BET proteins abundance confer resistance to the BET inhibitor in multiple cancer types (Dai et al., 2017; Zhang et al., 2017).

Neuroblastoma (NB) is the most common extracranial solid tumor in children. Amplification of *MYCN* is the important driver of high-risk neuroblastoma. Therefore, MYCN is considered a remarkable target for drug development in the treatment of *MYCN* amplified neuroblastoma (Huang and Weiss, 2013). BRD4, the well-studied member of the BET family proteins, plays an important role in *MYCN* expression *via* binding to acetylated histones at the super enhancer sites to regulate the transcription of *MYCN* and *c-Myc* (Henssen et al., 2016; Taniguchi, 2016). JQ1, the first developed BET inhibitor, could efficiently suppress the expression of *MYCN* in neuroblastoma by inhibiting the binding of BRD4 with acetyl lysine of histone (Delmore et al., 2011). Importantly, the BET inhibitors, such as JQ1, I-BET726 and OTX015 exhibited efficient anti-tumor effect in NB by markedly inhibiting the growth of neuroblastoma cells and prolonging the survival time of tumor-bearing mice. Therefore, BET proteins are the potential therapeutic targets to combat *MYCN* amplified neuroblastoma by inhibiting the expression of *MYCN* and other related oncogenes.

Here, the critical function of BET proteins as epigenetic reader in cancer development and the research progress to target BET protein in neuroblastoma was complicated summarized and discussed.

### The basic role of bromodomain and extra-terminal protein as an epigenetic reader

Histone acetylation is a reversible biological process controlled by the acetylation “writer” histone acetyltransferases (HATs) that transfer the acetyl groups on histone tails and the “eraser” histone deacetylases (HDAC) that remove the acetyl groups from histone (Ito et al., 2011; Noguchi-Yachide, 2016). The BET family proteins are subfamily of bromodomain protein superfamily. The N-terminal of human BET family proteins contains two conserved bromodomains (BD1 and BD2) which can recognize acetylated histones and regulate gene transcription and the C-terminal contains an extra-terminal (ET) domain. Bromodomain includes four α spiral slices (αZ, αA, αB, and αC) and two rings (ZA and BC) (Vollmuth and Geyer, 2010; Tang et al., 2021). Together with α-helixes, the ring region form a hydrophobic cavity core to recognize acetyl-lysine (Shi and Vakoc, 2014; French, 2016). The BET family proteins consist of BRD2, BRD3, BRD4, and BRDT (Law et al., 2018; Carlson et al., 2019). BRD4 is the well-studied member of the BET family due to its overexpression or fusion with other genes in caner development and drug resistance. BRD4 can bind to acetyl lysine on super enhancers and promoter of histones and transcription factors, leading to BRD4 localization in chromosomes where they recruit other regulatory complexes to affect gene expression (Kulikowski et al., 2021). The regulatory complexes include the core positive transcription elongation factor b (P-TEFb) and Mediator (Shi and Vakoc, 2014; Mochizuki et al., 2021). P-TEFb, which is constituted CDK9 and cyclinT1, is an important transcription factor in the process of gene expression. The interaction with BRD4 dissociates P-TEFb from the 7SK/HEXIM complex and activates P-TEFb kinases (Jang et al., 2005; Yang et al., 2005). Subsequently, CDK9 phosphorylates C-terminal Ser2 of Pol II and forms a stable transcription elongation complex, which ultimately promotes the expression of many target oncogenes of BET family, such as MYC and Bcl2 (Zhu et al., 2016; Hogg et al., 2017; Yin et al., 2020). Moreover, enhancer RNAs (eRNA) such as PSA eRNA could affect the P-TEFb activation in different cancer types through CYCLIN T1/CDK9 complex (Schaukowitch et al., 2014; Zhao et al., 2016). BRD4 bromine domain not only binds acetylated histones, but also interacts with acetylated transcription factors (TFs) (Cheung et al., 2017). Roe *et al.* demonstrated that after TF was acetylated by p300, BRD4 could be recruited and located at a specific location to promote the transcription of TF (Roe et al., 2015). In addition, the ET domain of BRD4 is responsible for additional protein-protein interactions, making BETs become the function core to promote the formation of polymerized structure composed of recruited TFs and coactivators (Wang et al., 2021).

### Bromodomain and extra-terminal proteins are therapeutic targets in cancer

The therapeutic agents targeting chromatin “writers” and “erasers” have been successfully developed. For example, HDAC inhibitors showed positive effect on tumor cell inhibition, but their clinical application is limited due to side toxic effect. Given that many downstream targets of BET proteins are pro-oncogenes, therefore, dysregulation of BET is closely correlated with cancer development and BET proteins have become novel drug targets. In 2010, the small molecule BET inhibitor JQ1 (Filippakopoulos et al., 2010) and I-BET (GSK525762A) was developed (Nicodeme et al., 2010). They have high affinity to the bromodomain of BET family members and could competitively bind to acetylated peptides, thus block the interaction of BET protein with chromatin. The BET inhibitor I-BET726 developed by GSK has high affinity and selectivity to BRD2, BRD3 and BRD4 (Gosmini et al., 2014). In 2012, the I-BET762 was approved for phase I clinical trials. In 2013, Picaud *et al.* found that PFI-1 inhibitors were acetyl lysine mimics and could replace acetyl lysine to bind to BRD2 and BRD4, thereby inhibiting the recruitment of BET protein to chromatin, down-regulating the expression of *MYC* and Aurora B kinases (Alqahtani et al., 2019). It was reported that RVX-208 can bind to BET proteins, especially BRD4, and regulate *ApoA-I* expression which playing a therapeutic role in atherosclerosis (Vertessy et al., 2013). The BET inhibitor OTX015 is a selective inhibitor for BRD2, BRD3, and BRD4 by inhibiting their binding to AcH4. In mature B-cell lymphoid tumors, OTX015 showed positive inhibitory effect by regulating *MYC* and *E2F1* genes expression and NF-κB/JAK signaling pathways (Boi et al., 2015). ABBV-075 is a novel BET inhibitor exhibited significant inhibitory effect on tumor growth in Kasumi-1 cells xenograft mice model (McDaniel et al., 2017) and prostate cancer which provides a new option for therapeutic treatment of CRPC patients (Faivre et al., 2017).

Interestingly, it has been reported that BD1 and BD2 domains can also regulate different gene sets by specifically recognizing acetylated lysine residues (Westermann et al., 2008; Gilan et al., 2020; Fu et al., 2021). For example, BRD4-BD2 could recruit transcription factor TWIST and BRD4-BD1 is responsible for the binding to chromatin (Shi et al., 2014). BRDT-BD1, but not BRDT-BD2, is necessary for spermatogenesis (Shang et al., 2007). BRD4-BD1 covalent inhibitors, such as Olinone, ZL0580, MS436, inhibit the transcription of BET target genes to retard the proliferation of tumor cells (Tang et al., 2021). However, the ABBV-744, an efficient BRD4-BD2 domain inhibitor, inhibits AR-dependent transcription of genes in prostate cancer xenograft model (Faivre et al., 2020).

Aberrant *c-Myc* expression is frequently found in inflammation and cancer (Leal et al., 2017; White et al., 2019) and MYC is thus considered a promising therapeutic target but it is also an “undruggable” target. Mechanismly, BET inhibitors exert their anti-tumor effect mainly through the inhibition of MYC, a downstream gene of BRD4, in many cancer types. In myeloma cell line MM1.S, JQ1 treatment results in downregulation of *MYC* expression and inhibition of cell proliferation (French, 2016). JQ1 and OTX015 treatment reduced *c-MYC* expression and led to cell growth inhibition, cell cycle arrest and apoptosis in acute leukemia cells and patient-derived leukemic cells (Coude et al., 2015). In addition, BET inhibitors are also effective in inhibiting medulloblastoma (Henssen et al., 2013) and hepatocellular carcinoma (Li et al., 2016) by downregulating *MYC* expression.

However, BET inhibitor could inhibit tumorigenesis in an MYC independent manner. NUT midline carcinoma (NMC) is caused by translocation-derived fusion proteins BRD4-NUT or BRD3-NUT. JQ1 treatment can detach BRD4 and BRD4-NUT from chromatin (Filippakopoulos et al., 2010; French, 2016). In addition, I-BET151, OTX015 and JQ1 inhibit the interaction between BRD4 and the acetylated NF-κB subunit RelA at lysine 310 site, which subsequently reduced the transcriptional activation of NF-κB (Wu et al., 2013; Alqahtani et al., 2019). After androgen ablation, androgen receptor (AR) signal is the main driver for the development of castration-resistant prostate cancer (CRPC), JQ1 treatment inhibited the interaction between BRD4 and AR and subsequently hindered AR-mediated gene transcription (Asangani et al., 2014; Sahai et al., 2016). Furthermore, JQ1 showed markedly inhibitory effect on lung adenocarcinoma through downregulating *FOSL1* and its targets (Lockwood et al., 2012). Moreover, JQ1 also demonstrated anti-tumor effects *via* inhibiting the expression of Forkhead box protein M1 (FoxM1) in ovarian cancer (Zhang et al., 2016) and aurora A kinase in triple-negative breast cancer (Sahni et al., 2016), respectively. Importantly, Donati et al. complicated summarized and discussed the key BRD4 target genes in normal and tumor cells such as embryonic cells, somatic cells, neuron cells, cardiac cells and cancer cells. The genes are related to osteogenesis, abiogenesis, myogenesis, NF-kB signaling pathway, estrogen and androgen receptor target genes including TNFα, IL8, GREB1, TFF1, PSA, HOXB13, CAMKK2, CCND1, MAPK8/10, and FOSL1 (Donati et al., 2018). Therefore, exploring more BRD4 downstream targets will help to understand the underlying mechanisms of the oncogenic function of BRD4 and other BET family members.

Altogether, BET inhibitors play important roles in cancer treatment by downregulating the expression of various BET target oncogenes (Table 1).

**TABLE 1:** BET inhibitors are used in multiple cancer types.

BET inhibitor	Cancer type	Target(s)	Mechanism	References
JQ1	MM	BRD2/3/4	Downregulation of *MYC*	French, (2016)
	Medulloblastoma	BRD2/3/4	Downregulation of *MYC*	Henssen et al. (2013)
	Hepatocellular carcinoma	BRD2/3/4	Downregulation of *MYC*	Li et al. (2016)
	NMC	BRD4	Evict BRD4 and BRD4-NUT from chromatin	French (2016), Filippakopoulos et al. (2010)
	CRPC	BRD2/3/4	Inhibit AR to activate its targeted genes	Asangani et al., 2014
				Sahai et al. (2016)
	Lung adenocarcinomas	BRD2/3/4	Downregulation of *FOSL1*	Lockwood et al. (2012)
	Ovarian cancer	BRD2/3/4	Disruption of FoxM1 pathway	Zhang et al. (2016)
	Triple-negative Breast Cancers	BRD2/3/4	Suppression of Aurora Kinase	Sahni et al. (2016)
RVX-208	Atherosclerosis	BRD4	ApoA-I	Vertessy et al. (2013)
ABBV-075	Prostate cancer	BRD4	inhibition of *c-Myc* expression	McDaniel et al. (2017)
Olinone, ZL0580, MS436	oligodendroglioma, HIV	BRD4-BD1	MYC, BCL2	Tang et al. (2021)
ABBV-744	Prostate cancer	BRD4-BD2	AR-dependent transcription of genes	Faivre et al. (2020)
JQ1/I-BET 762/OTX015	Neuroblastoma	BRD2/3/4	Downregulation of the expression of *MYCN* and *Bcl-2*	Puissant et al. (2013), Wyce et al. (2013), Henssen et al. (2016)
OTX015	Primary acute leukemia	BRD2/3/4	Downregulation of *MYC*, Upregulation of *HEXIM1*	Coude et al. (2015)

MM, multiple myeloma; NMC, NUT, midline carcinoma; CRPC, Castration-resistant prostate cancer.

### The critical role of MYCN in neuroblastoma

Neuroblastoma (NB), the most common extracranial solid tumor in children and newborns, originates from neural crest progenitors especially the adrenal glands (Brodeur, 2003; Zafar et al., 2021). The 5-years survival rate of patients with high-risk neuroblastoma are less than 50% (London et al., 2005). MYC-family transcription factors, including c-Myc, N-Myc and L-Myc, regulate cell proliferation and survival in multiple cancer types (Albihn et al., 2010). Among them, *MYCN* amplification accounts for 20% of primary neuroblastoma and it is closely associated with advanced NB and resistance to treatment (Hansford et al., 2004; Westermann et al., 2008). Moreover, *MYCN* amplification occurs in 40%–50% of high-risk NB (Bell et al., 2010). However, the amplification of other MYC family members such as MYCL and MYC is infrequently observed in NB patients. Furthermore, the expression of *MYC* is inversely correlated with *MYCN* expression (Breit and Schwab, 1989). Importantly, in mice embryos, *Myc* is ubiquitously expressed through the developmental stages but *MYCN* strictly expressed in the hematopoietic stem cells and cells of developing nervous system (Zimmerman et al., 1986; Trumpp et al., 2001). The restricted expression profile of *MYCN* might be mirrored the human NB which arise from the undifferentiated neural crest cells. Transgenic mouse model indicated that dysregulation of *MYCN* expression in neural crest was sufficient to induce tumorigenesis (Weiss et al., 1997). Therefore, MYCN has been considered a strong predictor of poor prognosis and mortality and an attractive target for therapeutic intervention in high-risk neuroblastoma (Westermark et al., 2011).

Mechanismly, as a transcription factor, MYCN can regulate the expression of many target genes, thereby regulating the basic processes of cell proliferation, protein generation, apoptosis and differentiation (Eilers and Eisenman, 2008). For example, in *MYCN*-amplified neuroblastoma, MYCN can bind to the promoter of telomerase catalytic subunit TERT, up-regulate and activate the expression of *TERT* which is a key function of amplified *MYCN* (Nikiforov et al., 2002; Pugh et al., 2013). Importantly, MYCN can also inhibit the expression of many cell adhesion related genes and cell cycle negative regulators (Westermark et al., 2011). Studies have shown that downregulation of *MYCN* by RNA interference led to cell cycle arrest and induction of apoptosis (Westermark et al., 2011). In addition, blocking the upstream signaling pathway that can regulate *MYCN* expression and protein stability and targeting the key downstream targets of MYCN may be another method to attenuate the effect of MYCN (Bell et al., 2010; Gustafson and Weiss, 2010). For example, inhibiting PI3K or mTOR can reduce the protein level of MYCN due to that PI3K/Akt/mTOR can maintain the stability of MYCN (Segerstrom et al., 2011).

Therefore, MYCN plays an important role in promoting the development of NB by activating or upregulating the multiple downstream genes.

### Targeting bromodomain and extra-terminal protein to inhibit neuroblastoma through regulating MYCN

Pan-cancer genome studies have shown that the mutation rate of genes in childhood cancer is significantly lower than that in adult cancer. Few recurrent mutations are detected in pediatric neuroblastoma which suggests that epigenetic disorders might play an important role in the development of childhood cancer (Iniguez et al., 2018). The result of chromatin immunoprecipitation (ChIP) indicated that BRD4 is enriched in *MYCN* promoter and enhancer regions to facilitate the expression of *MYCN*. Therefore, blocking the BET proteins by molecule inhibitors could be an efficiently therapeutic strategy to inhibit MYCN function. Currently, three BET small molecule inhibitors, JQ1, I-BET726, and OTX015, have been examined and showed significant inhibitory effect on the growth of neuroblastoma, which provides important support for the clinical application of BET inhibitors in the treatment of neuroblastoma patients. JQ1 treatment blocked the enrichment of BRD4 on the *MYCN* promoter region, then downregulated the transcription activity of *MYCN* and the expression of *MYCN* target genes. Furthermore, the transcription activity of *MDM2* was also reduced upon MYCN inhibition which subsequently increased *p53* expression that eventually led to apoptosis (Chen et al., 2010; Mazar et al., 2020). Moreover, JQ1 could induce cell cycle arrest in *MYCN*-amplified neuroblastoma *in vitro* and block tumor growth *in vivo* by inhibiting *MYCN* expression (Puissant et al., 2013). Lee et al. demonstrated that JQ1 treatment significantly increased the expression of key differentiation markers in the NB cell lines, indicating that the therapeutic effect of JQ1 could be achieved by promoting the differentiation of neuroblastoma (Lee et al., 2015). I-BET726, a novel BET small molecule inhibitor, binds to the hydrophobic cavity of BET family proteins to block the interaction of BRD4 and acetylated histone which downregulates *MYCN* and *BCL2* expression to retard NB cell growth, promote differentiation and apoptosis (Galderisi et al., 1999; Jiang et al., 2011; Wyce et al., 2013). Henssen et al. found that *MYCN*-amplified neuroblastoma cell lines were more sensitive to OTX015 treatment than those *MYCN* non-amplification NB cells in their *MYCN*-driven neuroblastoma xenograft model (Henssen et al., 2016).

**FIGURE 1:**
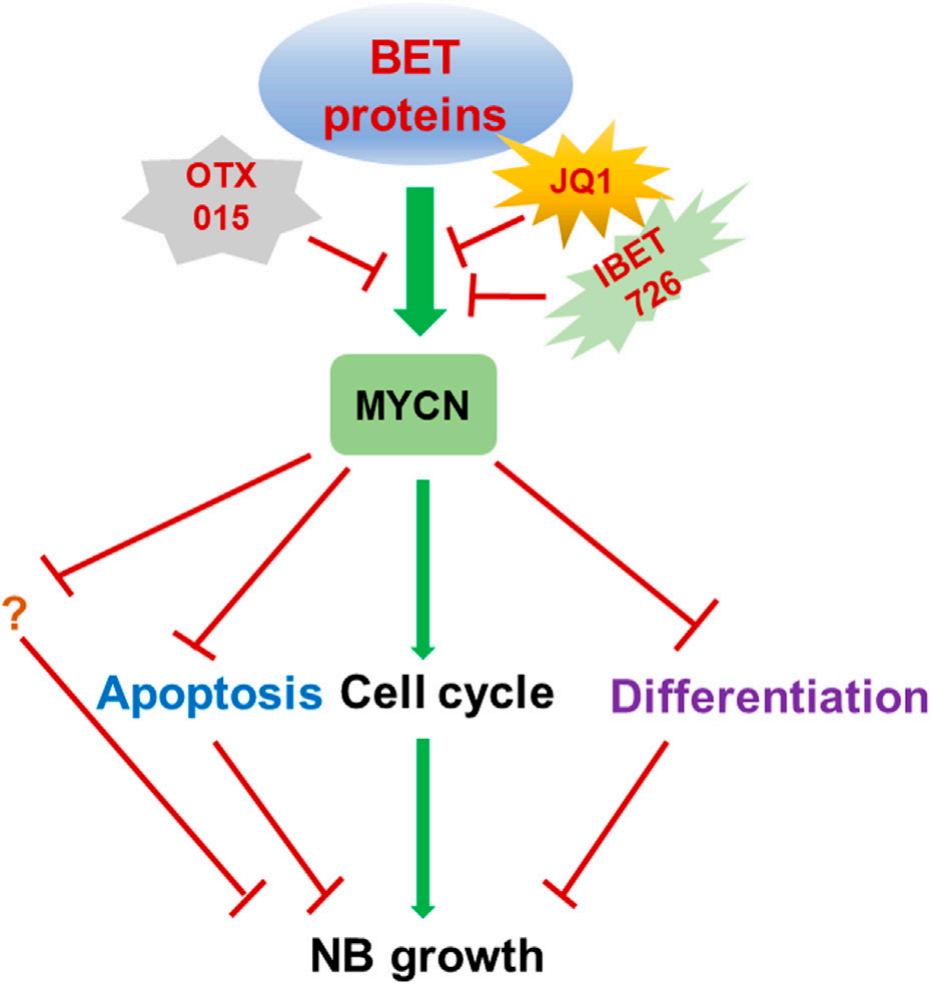
Targeting BET proteins inhibits NB growth through MYCN. Targeting BET proteins by different BET protein inhibitors such as JQ1, i-BET726 and OTX015 is therapeutic strategies which are widely tested in multiple cancer types including *MYCN* amplified NB. BET inhibitor treatment could induce cell cycle inhibition, apoptosis and differentiation by regulating the MYCN target genes.

Altogether, targeting BET proteins such as BRD4 by small molecule inhibitors of BET proteins is potent therapeutic strategy to efficiently inhibit the high-risk NB by downregulating *MYCN* expression (Liu et al., 2016) (Figure 1).

### Potential molecular mechanisms of resistance to bromodomain and extra-terminal inhibitor in neuroblastoma

It was well known that, resistance to chemotherapy are frequently found in multiple cancer types and chemotherapy strategies. Therefore, the drug resistance of NB tumor cells to BET inhibitors is also occurred which hampers the clinical application of the BET inhibitors on NB patients (Felgenhauer et al., 2018; Iniguez et al., 2018). For example, the abnormal activation of ERK1/2 signal in JQ1 resistant neuroblastoma cells attenuated the antitumor role of BET inhibitor by stabilizing MYCN protein (Liu et al., 2021). Furthermore, the PI3K pathway activation is another resistance mechanism to BET inhibitors in NB cells (Iniguez et al., 2018). Anastasia Wyce et al. found the expression level of BCL2 might affect the sensitivity of NB cells to BET inhibitor GSK1324726A (Wyce et al., 2013). Moreover, the activation of *NOTCH*1 and the expression of *GNAS*, *MDM2*, and *NF2* might be the predictors of resistance to BET inhibitors (Puissant et al., 2013). Although the gene expression signatures related with sensitivity to BET inhibitors in NB cells have been examined, but further investigation for their effect in clinical trials are still needed (Stathis and Bertoni, 2018).

### Combined therapeutic strategies applied in neuroblastoma therapy

**FIGURE 2:**
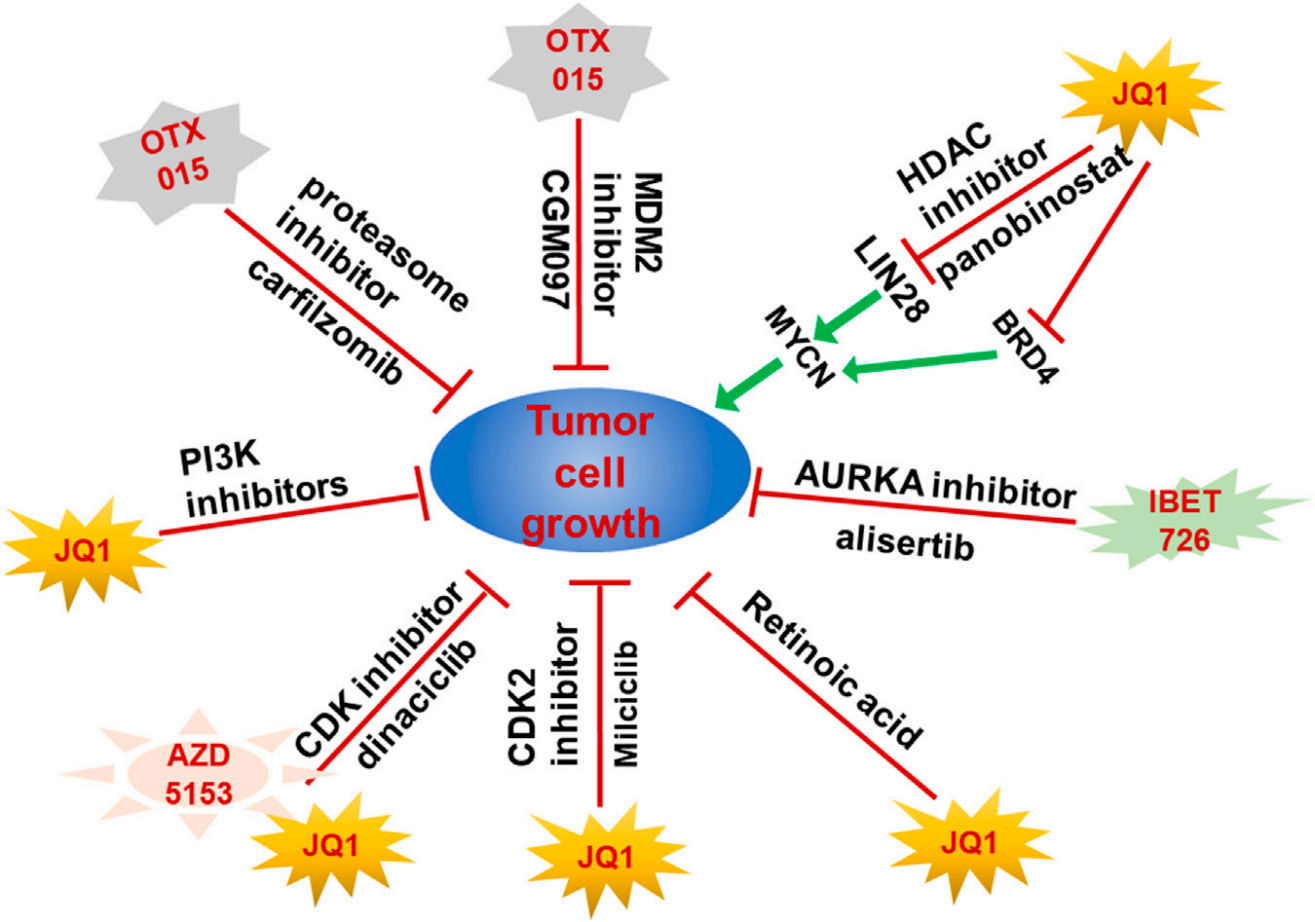
Combination of BET proteins inhibitors and other drugs achieved better therapeutic effect on NB growth. Resistance to BET proteins inhibitors hampered their clinical application. Combination of BET protein inhibitors with other small molecular inhibitors exhibited synergistic inhibition effect on NB tumors by targeting both MYCN and other genes upregulated or activated in resistance NB tumors.

The emerging resistance to BET inhibitors affects the therapeutic effects on cancer patients and the activation of key oncogenes or inactivation of tumor suppressors are important mechanisms of BET inhibitor resistance. Therefore, it is important to explore the new therapeutic strategies by combining BET inhibitors with other drugs or methods. However, although the combination for BET and MEK inhibition showed markedly synergistic effect on tumor cells growth and apoptosis in multiple NB cell lines, but their synergistic effect on tumor growth *in vivo* is limited (Healy et al., 2020). In 2016, Shahbazi et al. found that JQ1 could play a synergistic role with HDAC inhibitor panobinostat to significantly decrease the expression of *MYCN* by reducing the transcription of *LIN28B* in their *in vivo* and *in vitro* models (Shahbazi et al., 2016). Moreover, the combined application of BRD4 inhibitor I-BET151 and AURKA inhibitor alisertib significantly inhibited the neuroblastoma cells growth and dramatically prolonged the survival time of neuroblastoma xenograft mice (Felgenhauer et al., 2018). Furthermore, PI3K inhibitors could overcome the resistance of NGP cell to JQ1 which indicated that PI3K inhibitors and BET inhibitors have a strong synergistic effect (Iniguez et al., 2018). Interestingly, the proteasome inhibitor carfilzomib showed a synergistic anti-tumor effect with BET inhibitor OTX015. Since OTX015 is currently in phase II clinical trials and carfilzomib is an approved anti-tumor drug, the combination of OTX015 and carfilzomib is likely to be the first targeted therapy in the clinical trials for patients with TERT-rearrangement neuroblastoma (Chen et al., 2021). Moreover, JQ1 and CDK inhibitor dinaciclib showed synergistic effect on the induction of cytotoxicity in *MYCN* amplified NB cells but the combination of AZD5153 and dinaciclib reduced the tumor size in mice models *in vivo* through increasing the tumor necrosis and lymphocyte infiltration (Wood et al., 2021). Specifically, JQ1 synergized with CDK2 inhibitor Milciclib which has been used in clinical trials to induce apoptosis and inhibit *MYCN* amplified NB cell growth by downregulating the MYC target genes (Bolin et al., 2018). Although the retinoic acids (RAs) were applied in the high-risk NB differentiation therapy but the effectiveness is limited, low dose of JQ1 and RA could synergistically inhibit NB cells proliferation and induce differentiation which indicated BET inhibitor and RA might be a combination therapy in combating NB (Alleboina et al., 2021). Importantly, p53 inactivation is frequently observed in the NB tumors after relapse, therefore, the combination of MDM2 inhibitor CGM097 and BET inhibitor OTX015 exhibited a synergistic inhibition of NB cell growth by activating *p53* and decreasing expression of *MYC* family proteins (Maser et al., 2020) (Figure 2).

Except the BET inhibitors which recognize and bind to BET proteins to block the interaction of BRD4 with acetylated histone, the selective BET inhibitors which specifically bind to BD domain of BRD4 are also developed (Shi et al., 2014). In 2020, Slavish et al. demonstrated that SJ432, a BD2 Selective inhibitors, could reduce the MYC protein level in neuroblastoma cell line. Furthermore, the NB mouse models treated with SJ432 showed a smaller tumor volume and longer survival time than those mice treated with JQ1 (Slavish et al., 2020).

Therefore, the exploration of combined therapeutic strategy and novel selective inhibitors for BET proteins will help to efficiently combat the neuroblastoma in a more precise manner.

## Discussion

In this review, the basic function of BET protein in regulating gene expression as epigenetic “reader” and the therapeutic effect of BET inhibitors on cancer development is complicated discussed. More importantly, we also emphasized the critical role of BET inhibitors in combating neuroblastoma through regulating MYCN.

It is well known that patients with high-risk NB have a less than 50% 5-years survival rate (Kang et al., 2006). The amplification of various oncogenes favorable NB development are commonly observed in high-risk NB patients. Notably, the amplification of *MYCN* is found in 20% of high-risk NB patients and amplification of *MYCN* is considered the best genetic marker and therapeutic target for high-risk NB (Weiss et al., 1997; Valentijn et al., 2012). However, MYCN is thought to be a “undruggable” target which make it impossible to directly target MYCN proteins. Therefore, inhibiting *MYCN* expression by blocking *MYCN* transcription regulators such as the epigenetic reader proteins is an important therapeutic strategy. BET proteins, the epigenetic readers, contain two bromodomains which can recognize acetylated histones and regulate gene transcription (French, 2016). BRD4 plays an important role in *MYCN* expression. BRD4 can bind to acetylated histones at the super enhancer sites and regulate the transcription of *MYCN* and *c-Myc* (Henssen et al., 2016). The three BET inhibitors, JQ1, I-BET726 and OTX015 exhibited efficiently antitumor effect in NB in the *in vitro* cells and *in vivo* xenograft models. However, the results of phase I clinical trials of BET inhibitors in human cancer patients did not show ideal therapeutic benefits. Moreover, the expression level of targeted genes might be an important factor to induce drug resistance. It was reported that the triple-negative breast cancer (TNBC) cell lines with higher expression level of *MYCN* are more sensitive to BET inhibitor (Schafer et al., 2020). Furthermore, Alexandre *et al.* conducted a high-throughput, cell-based screening of different NB cancer cell lines, and they found the *MYCN* amplification was a strong predictor of sensitivity to JQ1 treatment (Puissant et al., 2013) which indicated a marked correlation between the *MYCN* amplification status and the sensitivity to BET inhibitor.

Given that the side effects and drug resistance might be an important obstacle for the clinical utilization of BET inhibitors (Bechter and Schoffski, 2020). The combination of BET inhibitors with other targeted and epigenetic therapies might be an alternative therapeutic strategy to conquer the side effect of single administration of BET inhibitor or drug resistance. The combination of BET inhibition with PI3K/AKT/PTEN pathway inhibition can overcome the drug resistance caused by single usage of the two drugs (Alqahtani et al., 2019). In addition, BRD4 can be inhibited together with other epigenetic regulators, such as HDAC, to achieve the best anti-cancer effect (Mohammad et al., 2019). It should be noted that the overlapping toxicity should be carefully considered during the application of combination therapy. Recent study showed that combined BET and MEK inhibition showed synergistic effect in inhibiting multiple NB cells growth and survival *in vitro* but the antitumor activity *in vivo* was limited which is possibly caused by the expression level of *MYCN* and other oncogenes such as *NF1* (Healy et al., 2020). More importantly, the Proteolysis Targeting Chimeric (PROTAC) technology was developed to promote BRD4 for proteasome mediated degradation which show different mechanisms from the BET protein inhibitors. Winter et al. created a bifunctional JQ1 molecule fused with thalidomide, called dBET which can specifically target BET protein for E3 ligase mediated degradation, resulting in a higher level of apoptosis in primary AML cells than those treated with JQ1 alone (Winter et al., 2015). Importantly, Li et al. found the PROTAC ARV-825 exhibited profound anti-tumor activity in NB cell lines and NB xenograft mice models by inhibiting the expression of *MYCN* or *c-Myc* (Li et al., 2020). Furthermore, BET degraders caused more marked cytotoxic effect on multiple cancer types by degrading the BET proteins than the BET protein inhibitors (Lu et al., 2015; Raina et al., 2016; Winter et al., 2017). Whether the BET degraders could more efficiently inhibit the NB development than BET protein inhibitors or are valuable in combating drug resistance in NB need further in deep investigation.

In summary, BET proteins are the important upstream regulators of MYCN and inhibiting BET proteins by the BET inhibitors alone or combined with other therapeutic strategies might be efficient methods to inhibit NB development by attenuating the expression of *MYCN*. In addition, exploring the new inhibitors of BET family proteins and the novel combined therapeutic strategies have made valuable contributions to the treatment of NB patients.
